# Clinical narrative competence and humanistic care ability of nurses in assisted reproductive technology: a cross-sectional study

**DOI:** 10.1186/s12912-024-01791-6

**Published:** 2024-02-15

**Authors:** Fengyi Mo, Xiaorui Hu, Qing Ma, Lanfeng Xing

**Affiliations:** Department of Reproductive Endocrinology, Women’s Hospital, School of Medicine, Zhejiang University, Hangzhou, People’s Republic of China

**Keywords:** Assisted reproductive technology, Nurse, Clinical narrative competence, Humanistic caring, Cross-sectional study

## Abstract

**Background:**

Growing focus on patient-centred care emphasizes humanistic skills and clinical narrative competence in nursing, particularly in assisted reproductive nursing. However, there is limited evidence to suggest the levels of nurse’ clinical narrative competence and humanistic care ability. This study aimed to investigate the clinical narrative competence and humanistic care ability of nurse specialists in assisted reproductive technology (ART) in China.

**Methods:**

This cross-sectional study included nurses who obtained the ART specialist nurse certificate after nurse training in Zhejiang province assisted reproductive technology specialist nurse training base between 2017 and 2022. A demographic questionnaire, the Caring Ability Inventory (CAI) and Narrative Competence Scale (NCS) were used for data collection. Multivariate linear regression analysis was used to explore risk factors.

**Results:**

A total of 122 participants (120 females, with a mean age of 33.35 ± 5.00 years) were included (response rate = 82.43%). NCS score and CAI score was 143.39 ± 19.24 (range: 27–189) and 198.42 ± 19.51 (range: 37–259) among nurse specialists in assisted reproductive technology, respectively. Multivariate linear regression analysis indicated that professional title (β *=* 20.003, 95%CI: 3.271–36.735, P *=* 0.020), and the CAI (β *=* 0.342, 95%CI: 0.180–0.540, P *<* 0.001) was independently associated with NCS. Head Nurse/ Team Leader/ Clinical Faculty had significantly higher CAI score than nurse (*P* = 0.006).

**Conclusions:**

The clinical narrative competence and caring ability of nurse specialists in assisted reproductive technology was considered sufficient. Professional titles and work position were associated with clinical narrative competence. Enhancing clinical narrative competence can be considered as an effective strategy for promoting humanistic care ability.

**Trial registration:**

Not applicable.

**Supplementary Information:**

The online version contains supplementary material available at 10.1186/s12912-024-01791-6.

## Background

Assisted reproductive technology (ART) refers to medical procedures used primarily to address infertility. These include in vitro fertilization (IVF), intracytoplasmic sperm injection (ICSI), cryopreservation of gametes or embryos, and the use of fertility medication [1, 2]. Since ART was first successfully applied in 1978, it has advanced remarkably and is widely-used in clinics for the treatment of infertility [3]. As of December 2022, a total of 559 reproductive medical centres have approved ART in China, and carried more than 1,000,000 reproductive treatment cycles yearly. The rapid development of ART has placed greater professional requirements on assisted reproductive nurses (ARN) [4]. ARN are pivotal in ART, influencing patient outcomes and well-being through expert care and emotional support, crucial for successful treatment and holistic patient health [5, 6].

While ART can address many reproductive issues, it cannot guarantee pregnancy for all women and carries risks like birth defects, low birth weight, and preterm deliveries [7, 8]. Infertile couples bear the burden of medical interventions related to the diagnosis and treatment of ART. Couples may need to tolerate high stress levels, and they need to manage their behavioral, mental and emotional responses to infertility and ART procedure [9]. Also of concerns is the strong need for emotional, psychosocial and informational support in infertile couples, while focusing on technology development. This place high demands on the level of nurses’ humanistic abilities [10], focusing on the holistic well-being of patients undergoing ART. Providing humanistic care and psychological support, along with enhancing clinical narrative competence, becomes essential in ensuring comprehensive patient care in this sensitive medical field [9, 10]. Since the late twentieth century, medical humanities have been recognized as a crucial aspect of medical education [11]. This rise coincides with the increasing emphasis on patient-centred care and the value of humanistic qualities in nursing for practicing medical humanities. Bridging this is narrative medicine, a patient-centred approach enhancing humanistic engagement through narrative, serving as an effective tool for medical humanities [12–14]. However, the success of narrative medicine hinges on the clinical narrative competence of medical professionals, especially nurse specialists who are integral to nursing practice. Over the past two decades, narrative medicine programs have become widespread globally [15]. China has seen a surge in research on narrative medicine, with colleges increasingly incorporating narrative competency training into medical education [16–21]. The educational reform aimed to transition from lecture-based to practice-oriented teaching, yet the direct implementation of narrative medicine in clinical healthcare is still limited.

Although humanistic care has been emphasised and extensive efforts have been made in nursing education, the integration of humanistic care into clinical practice remains optimistic, especially in China [22]. Previous studies have reported that Chinese nursing students tend to possess a relatively high level of caring knowledge. However, they tend to lack certain abilities [23, 24]. To the best of our knowledge, the clinical narrative competence and humanistic care ability of nurses in ART has not been reported. More information about the nurses’ clinical narrative competence and humanistic care ability is needed to understand and improve training and caring strategies. Therefore, this study aimed to investigate the clinical narrative competence and humanistic care ability of nurse specialists in assisted reproductive technology in China.

## Methods

### Study design

A quantitative design based on a cross-sectional study was used.

### Participants

Nurses who obtained the ART specialist nurse certificates after undergoing training in Zhejiang province assisted reproductive technology specialist nurse training base between 2017 and 2022, were recruited for this study. The study adhered to strict inclusion criteria, selecting only nurses who completed this specific training. The exclusion criterion was a refusal to take part in the study or currently not practicing in the field of ART nursing.

### Ethics consideration

Ethical approval for this study was granted by the Institutional Review Board of Women’s Hospital, School of Medicine, Zhejiang University (Approval No. IRB-20220206-R). Written informed consents were obtained from all participants.

### Data collection tool

Nurse specialists in ART were asked bout demographic questionnaire, CAI and NCS.

### Demographic questionnaire

The demographic questionnaire collected participants’ information, including age, years of work experience, professional title, position, education level, gender, and the grade of their medical institution.

### Caring ability inventory (CAI)

The Caring Ability Inventory (CAI), originally developed by Nkongho [25], was utilized to assess humanistic caring abilities. This tool, comprising 37 items across three dimensions (cognition, courage, patience), employs a 7-point Likert scale, with higher scores indicating stronger caring abilities. The CAI, translated into various languages, had a Cronbach’s α coefficient of 0.849 in this study [26].

### Narrative competence scale (NCS)

The Narrative Competence Scale (NCS), created by Ma [27], evaluated medical staff’s narrative competence. It features 27 items in three dimensions (listening, understanding/responding, reflecting) and uses a 7-point Likert scale. Higher scores on this scale reflect greater narrative competence. The NCS showed a Cronbach’s α coefficient of 0.958 in this study, underscoring its reliability.

### Distribution and quality control

Nurses who completed ART specialist training in Zhejiang province were selected for this study. Participants first received and agreed to an electronic informed consent form before accessing the survey. The questionnaire, distributed via Questionnaire Star (https://www.wjx.cn/), was accessible through a web link, ensuring voluntary and anonymous participation. A dedicated team of two trained nurse research assistants oversaw the questionnaire’s promotion and distribution, rigorously checking responses for completeness and consistency. Responses with logical discrepancies, incomplete answers, or uniformity across items were deemed invalid.

### Method of data analysis

Data analysis was conducted using SPSS version 25.0 (IBM Corp., Armonk, NY, USA), with the Kolmogorov-Smirnov and Shapiro-Wilk tests assessing the normality of continuous variables. Continuous variables are represented as means ± standard deviation (SD), and categorical variables as frequencies and percentages. T-tests and ANOVA determined differences in CAI and NCS scores based on participant characteristics. Pearson’s correlation coefficient evaluated the CAI-NCS relationship. R software version 4.2.3 facilitated multivariate linear regression analysis, significant variables in the univariate analysis (*P* < 0.05) were included in the multivariate linear regression analysis. Two-sided *P* < 0.05 were considered statistically significant.

## Results

### Demographic characteristics

A total of 148 assisted reproductive nurses from 23 provinces or cities and 71 medical institutions, having completed assisted reproductive technology training were recruited, 26 participants were excluded due to incomplete questionnaires and 122 participants returned complete questionnaires (response rate = 82.43%). There were 120 (98.36%) females and with a mean age of 33.35 ± 5.00 years. Majority of participants were working in medical institutions with Grade III level A (84.43%), graduated with a undergraduate education level (90.98%). Working years ranged from 3 to 28 years, with a mean of 11.42 ± 5.42 years. More than half of the participants had a middle professional title (55.74%). 22.95% participants had a work position as head nurse, team leader, clinical faculty.

### Univariate analysis

The NCS score was 143.39 ± 19.24 ( range: 27–189) among nurse specialists in ART. Univariate analysis showed that the NCS score was significantly correlated with professional title (*P* < 0.01) and work position (*P* < 0.001). No statistical difference in the NCS was found among the different grades of medical institutions, working years, or education levels (*P* > 0.05) (Table 1). The CAI score was 198.42 ± 19.51 (range: 37–259) among nurse specialists in ART. Head Nurse/ Team Leader/ Clinical Faculty had significantly higher CAI score than nurse (*P* = 0.006) (Table 1).

**Table 1:** Clinical NCS and CAI among nurse specialist in ART (*N* = 122)

Variables	NCS	***p***	CAI	***p***
**Grade of medical institution**		0.364		0.488
Grade II Level-A (*n* = 9)	151.44 ± 20.43		197.00 ± 11.61	
Grade III Level-B (*n* = 10)	146.10 ± 12.16		205.50 ± 16.70	
Grade III Level-A (*n* = 103)	142.42 ± 19.65		197.85 ± 20.27	
**Working years**		0.087		0.800
< 10 Years (*n* = 54)	139.43 ± 17.71		198.67 ± 16.60	
10–15 Years (*n* = 39)	148.28 ± 17.79		196.87 ± 20.33	
≥ 16 Years (*n* = 29)	144.17 ± 22.63		200.03 ± 23.59	
**Professional Title**		0.001		0.110
Junior (*n* = 48)	139.27 ± 18.21		197.17 ± 15.97	
Middle (*n* = 68)	143.90 ± 18.61		197.87 ± 21.59	
Senior (*n* = 6)	170.50 ± 12.50		214.67 ± 15.27	
**Education Level**		0.061		0.777
Undergraduate (*n* = 111)	142.36 ± 18.94		198.58 ± 17.85	
Graduate (*n* = 11)	153.73 ± 20.12		196.82 ± 33.15	
**Work position**		< 0.001		0.006
Nurse (*n* = 94)	140.11 ± 18.11		195.78 ± 19.61	
Head Nurse/ Team Leader/ Clinical Faculty (*n* = 28)	154.39 ± 19.14		207.29 ± 16.59	

NCS: Narrative Competence Scale, CAI: Caring Ability Inventory

### Correlation analysis

The NCS was positively correlated with the CAI in terms of both total scores (*r* = 0.417, *P* < 0.01), and cognitive dimensions (*r* = 0.366, *P* < 0.01), courage dimensions (*r* = 0.239, *P* < 0.01), and patience dimensions (*r* = 0.251, *P* < 0.01), based on Pearson’s correlation (Table 2).

**Table 2:** Correlation analysis between NCS and CAI among nurse specialist in ART (*N* = 122)

Variables	NCS	CAI	Cognitive	Courage	Patience
NCS	1				
CAI	0.417**	1			
Cognitive	0.366**	0.712**	1		
Courage	0.239**	0.683**	0.066	1	
Patience	0.251**	0.574**	0.664**	-0.077	1

NCS: Narrative Competence Scale, CAI: Caring Ability Inventory. ** *p* < 0.01

### Multivariate linear analysis

Multivariate linear regression analysis suggested that professional title (β = 20.003, 95%CI: 3.271–36.735, *P* = 0.020), and the CAI (β = 0.342, 95%CI: 0.180–0.540, *P* < 0.001) was independently associated with NCS (Table 3).

**Table 3:** Mulvariate linear regression analysis of clinical narrative competence among nurse specialist in ART

	Multivariate			
Variables	*β*	Coefficient (95%CI)		***P***
**Professional title**				
Junior (*n* = 48)	Ref			
Middle (*n* = 68)	2.845	-3.925	9.615	0.407
Senior (*n* = 6)	20.003	3.271	36.735	0.020
**Work position**				
Nurse (*n* = 94)	Ref			
Head Nurse/Team Leader/Clinical Faculty (*n* = 28)	5.353	-3.309	14.015	0.226
CAI score	0.342	0.180	0.504	< 0.001

CAI: Caring Ability Inventory. SE = standard error of the unstandardized coefficient; *β =* standardized coefficient; t = validity coefficient of the regression. (*R*^*2*^ = 0.256, adjust *R*^*2*^ = 0.231, *F = 10.07*, *p* < 0.001)

## Discussion

This study found that the clinical narrative competence and caring ability of nurse specialists in assisted reproductive technology was considered sufficient. The NCS was positively correlated with the CAI. Professional titles and work position were associated with clinical narrative competence. Head Nurse/ Team Leader/ Clinical Faculty had significantly higher CAI score than nurse. This study may provide a foundation for future studies and interventions aimed at enhancing the skills and competencies of nurse specialists.

To adapt to the rapid growth of reproductive medicine, Zhejiang Province established its first training base for ART-specialised nurses in China in 2017. Between 2017 and 2022, a total of 148 trainees from 23 provinces or cities and 71 medical institutions obtained certification as specialised nurses after completing theoretical training, clinical practice training, assessment, and qualification certification. In addition to being senior practitioners of clinical nursing, ART nurse specialists not only undertake the whole process of nursing, technical implementation [28], and surgical cooperation for patients receiving ART treatment [29, 30], but are also responsible for providing instructions, patient education, therapeutic emotional support, explaining test results, decision-making consultation, follow-up, and other work [31, 32]. Due to the obvious negative emotions of infertile couples, such as anxiety, depression, isolation, and family and social tension [33–35], this ability is of great importance for nurse specialists in ART in terms of providing understanding, empathy, comfort, and counselling to patients. It has been shown that in the treatment of infertile couples, emotional, psychological and informational support can significantly alleviate patients’ adverse emotions, thereby improving the outcome of ART treatment [36, 37]. However, no research has focused on reporting the clinical narrative competence or humanistic care abilities of nurse specialists.

In this study, the NCS score was 143.39 ± 19.24 among nurse specialist in ART, which was lower than the 153.97 ± 15.15 among nurses from 14 general hospitals in the Jiangsu Province [38]. The above results are consistent with those of Zheng et al., possibly due to the fact that the nurses in this study were from different medical institutions in multivariate provinces and cities, with attention to narrative medicine varying greatly [39]. As a novel subject, narrative medicine has established its essential position in clinical medicine, and some colleges and universities, such as Peking Union Medical College and Southern Medical University, offer public elective courses in narrative medicine [40]. However, narrative medical education has not been included in the standardised training of nursing staff or nurse specialists; thus, the awareness of narrative medicine in clinical settings remains low.

Nursing practice in ART makes a significant contribution to patient care. The specialised role of the nurse practicing in the ART arena is continually evolving and expanding. Despite this, nurse specialists in ART have not been established in developed countries, such as the United States [41], Canada [42], the United Kingdom [43] and Japan [44]. The education, contextual knowledge and skills integral to this area of practice have not been clearly articulated, and there are significant differences and flexibility in the role functions undertaken by nurses in reproductive centres. However, reproductive medical staffs nowadays put more attention on technical, which favors healing over caring. The evaluation of nursing quality and professional development are largely dependent on the ability of ART nurses to articulate their practice, such as experience, knowledge, and procedural skill level, rather than humanistic practice or patient-centred functions. Our study provides valuable insights, the level of clinical narrative competence and caring ability of domestic nurse specialists in ART were sufficient, and this provides a foundation for advanced humanistic practice and narrative implementation in the near future.

This study found a significant association between clinical narrative competence and the CAI, which is consistent with the findings of Cui et al. [48]. The humanistic care ability of nursing staff is not innate, and its professional characteristics, such as caring emotions, social responsibility, humanitarian thought, values, and knowledge, need to be trained and strengthened by in-service education and clinical practice. Clinical humanistic care training programs mostly cover concept introduction, listening and communication skills, case analysis, simulation drilling, nursing etiquette, and other content [49, 50]. Its connotations are particularly consistent with the core elements of empathy and reflection in clinical narrative competence, which can effectively enhance the humanistic literacy and perception abilities of nursing staff. The results suggest that clinical narrative training in improving humanistic care ability should be explored and popularised in the nurse specialist training system.

The multivariate linear regression analysis indicated that nurse specialists with higher professional titles had higher NCS scores. The possible reasons for this might be that they had richer clinical work experience and stronger comprehensive quality ability while undertaking nursing work, to a greater extent, among patients with critical illness or difficulty communicating. In addition, they might have better abilities in communication, emotional management, observation, and coping 11, and could therefore pay more attention to the ideas and demands of patients, thus providing humanistic care. The results of this study have important implications for nursing education providers and nurse managers. The implication for nurse educators is enhancing clinical narrative training can be considered as an effective strategy for promoting humanistic care ability. For managers, it is important to understand the different characteristics of nurse specialists in ART to support humanistic caring transition from ability to practice, and thus ultimately facilitate improvement in quality of ART care.

This study had several limitations. First, it used self-reported data from an online application. This method is prone to potential recall or social desirability bias, which may lead to underreporting or overreporting of the outcomes. Second, the data were collected from participants selected through convenience sampling at a single training base of nurse specialists in ART; although the participants came from 71 representative medical institutions, the sample size was not particularly large, and it was not a multi-centre study. Therefore, the generalisability of the findings may be limited. Future large-scale national studies are required to confirm these findings.

## Conclusions

The clinical narrative competence and caring ability of nurse specialists in assisted reproductive technology was considered sufficient. Professional titles and work position were associated with clinical narrative competence. Enhancing clinical narrative competence can be considered as an effective strategy for promoting humanistic care ability and the promotion of a supportive training approach is important for senior staff and nurses.

### Electronic supplementary material

Below is the link to the electronic supplementary material.

Supplementary Material 1

## Data Availability

All data generated or analysed during this study are included in this published article.

## Abbreviations

CAI: Caring Ability Inventory
NCS: Narrative Competence Scale
ART: Assisted reproductive technology
IVF: In vitro fertilization
ICSI: Intracytoplasmic sperm injection
ARN: Assisted reproductive nurses
