# Supplementary material for: Clinical narrative competence and humanistic care ability of nurses in assisted reproductive technology: a cross-sectional study
Source: BMC Nurs. 2024 Feb 15;23:119. doi: 10.1186/s12912-024-01791-6 (PMC10870622; doi:10.1186/s12912-024-01791-6)
Supplement: Supplementary file 1 — Supplementary Material 1 [file 12912_2024_1791_MOESM1_ESM.docx]

**Questionnaire**

1.Demographic Questionnaire

| Name |  | Age |  |
| --- | --- | --- | --- |
| Gender |  | Working Years |  |
| Professional Title | □Junior □Middle □Senior □Other ________ | | |
| Education Level | □Undergraduate □Graduate □Other _______ | | |
| Affiliation |  | | |
| Medical Institution Grade | □Grade II Level-A □Grade II Level-B  □Grade III Level-A □Grade III Level-B  □Other ________________ | | |
| Work Position | □None □Head Nurse  □Team Leader □Clinical Faculty  □Other ________________ | | |
| Address |  | | |
| E-mail |  | | |
| Phone Number |  | | |
| Have you received any sort of humanistic training before? | □Yes □No  If yes, please answer the questions below:  □When did you finish the training ? ________________  □The organizer of the training ____________________  □Training content or subject ____________________  ______________________________________________ | | |

2.Caring Ability Inventory (CAI)

Please read each of the following statements and decide how well it re­flects your thoughts and feelings about other people in general. There is no right or wrong answer. Using the response scale, from 1 to 7, circle the degree to which you agree or disagree with each statement directly on the booklet. Please answer all questions.

1 2 3 4 5 6 7

Strongly Strongly

Disagree Agree

|  | Strongly Strongly  disagree agree |
| --- | --- |
| 1. I believe that learning takes time. | 1 2 3 4 5 6 7 |
| 2. Today is filled with opportunities. | 1 2 3 4 5 6 7 |
| 3. I usually say what I mean to others. | 1 2 3 4 5 6 7 |
| 4. There is very little I can do for a person who is helpless. | 1 2 3 4 5 6 7 |
| 5. I can see the need for change in myself. | 1 2 3 4 5 6 7 |
| 6. I am able to like people even if they don't like me. | 1 2 3 4 5 6 7 |
| 7. I understand people easily. | 1 2 3 4 5 6 7 |
| 8. I have seen enough in this world for what I need to know. | 1 2 3 4 5 6 7 |
| 9. I make the time to get to know other people. | 1 2 3 4 5 6 7 |
| 10. Sometimes I like to be involved, and sometimes I do not like being involved. | 1 2 3 4 5 6 7 |
| 11. There is nothing I can do to make life better. | 1 2 3 4 5 6 7 |
| 12. 1 feel uneasy knowing that another person depends on me. | 1 2 3 4 5 6 7 |
| 13. I do not like to go out of my way to help other people. | 1 2 3 4 5 6 7 |
| 14. In dealing with people, it is difficult to let my feelings show. | 1 2 3 4 5 6 7 |
| 15. It does not matter what I say, as long as I do the correct thing. | 1 2 3 4 5 6 7 |
| 16. I find it difficult to understand how the other person feels if I have not had similar experiences. | 1 2 3 4 5 6 7 |
| 17. I admire people who are calm, composed, and patient. | 1 2 3 4 5 6 7 |
| 18. I believe it is important to accept and respect the attitudes and feelings of others. | 1 2 3 4 5 6 7 |
| 19.People can count on me to do what I say I will. | 1 2 3 4 5 6 7 |
| 20.I believe that there is room for improvement. | 1 2 3 4 5 6 7 |
| 21.Good friends look after each other. | 1 2 3 4 5 6 7 |
| 22.I find meaning in every situation. | 1 2 3 4 5 6 7 |
| 23.I am afraid to let go of those I care for because I am afraid of what might happen to them. | 1 2 3 4 5 6 7 |
| 24.I like to offer encouragement to people. | 1 2 3 4 5 6 7 |
| 25.I do not like to make commitments beyond the present. | 1 2 3 4 5 6 7 |
| 26.I really like myself. | 1 2 3 4 5 6 7 |
| 27.I see strengths and weaknesses (limitations) in each individual. | 1 2 3 4 5 6 7 |
| 28.New experiences are usually frightening to me. | 1 2 3 4 5 6 7 |
| 29.I am afraid to be open and let others see who I am. | 1 2 3 4 5 6 7 |
| 30.I accept people just the way they are. | 1 2 3 4 5 6 7 |
| 31.When I care for someone else, I do not have to hide my feelings. | 1 2 3 4 5 6 7 |
| 32.I do not like to be asked for help. | 1 2 3 4 5 6 7 |
| 33.I can express my feelings to people in a warm and caring way. | 1 2 3 4 5 6 7 |
| 34.I like talking with people. | 1 2 3 4 5 6 7 |
| 35.I regard myself as sincere in my relationships with others. | 1 2 3 4 5 6 7 |
| 36.People need space (room, privacy) to think and feel. | 1 2 3 4 5 6 7 |
| 37.I can be approached by people at any time. | 1 2 3 4 5 6 7 |

3.Narrative Competence Scale (NCS)

Below you will find a set of statements followed by numbers from 1 to 7. Please read each statement carefully. After reading the statement, decide how well it describes you. If you strongly agree with a statement, circle 7. If, however, you strongly disagree with a statement, then circle 1. There are no right or wrong answers. Answer as honestly as possible. Please read and answer all items.

|  | Strongly Strongly  disagree agree |
| --- | --- |
| 1. I am able to promptly identify patients' narrative needs. | 1 2 3 4 5 6 7 |
| 2. When I realize that patients have narrative needs, I can do my best to meet them. | 1 2 3 4 5 6 7 |
| 3. I am able to listen attentively and carefully when patients narrate. | 1 2 3 4 5 6 7 |
| 4. I find it difficult to find appropriate topics to bring me closer to patients. | 1 2 3 4 5 6 7 |
| 5. I am able to cultivate a well-functioning relationship with patients in my daily work. | 1 2 3 4 5 6 7 |
| 6. I can find and understand the changes in the patient's voice, tone, and intonation during expression of narrative. | 1 2 3 4 5 6 7 |
| 7. I can find and understand the nonverbal behaviors of patients during expression of narrative (such as eyes, facial expressions, subtle movements, etc.) | 1 2 3 4 5 6 7 |
| 8. I do not criticize patients stories. | 1 2 3 4 5 6 7 |
| 9. I am able to mobilize the patients' narrative enthusiasm by using language, body movements, environmental atmosphere, etc. | 1 2 3 4 5 6 7 |
| 10. I am able to pay attention to my posture, movements, language and facial expressions during patients narrative. | 1 2 3 4 5 6 7 |
| 11.When patients narrate, I sometimes interrupt or forcefully guide them. | 1 2 3 4 5 6 7 |
| 12. I can encourage patients to narrate and to tell “their story” about how they experienced and handled their illness. | 1 2 3 4 5 6 7 |
| 13. I am able to objectively and comprehensively grasp the content of narratives. | 1 2 3 4 5 6 7 |
| 14. I can understand the deep meaning of patients story from the perspective of patients. | 1 2 3 4 5 6 7 |
| 15. I am able to organize the patient's cluttered narratives into a coherent sequence. | 1 2 3 4 5 6 7 |
| 16. I can find out the main issues related to the current situation from the patients narrative. | 1 2 3 4 5 6 7 |
| 17. I can help patients to overcome their difficulties. | 1 2 3 4 5 6 7 |
| 18. I am able to think about the role of medical staff and medical behaviors during patients narrative. | 1 2 3 4 5 6 7 |
| 19. I am able to identify my shortcomings and strengths when coping with patients narrative. | 1 2 3 4 5 6 7 |
| 20. I am able to make appropriate verbal or nonverbal responses to patients. | 1 2 3 4 5 6 7 |
| 21. I am able to avoiding harm to patients when responding to their narratives. | 1 2 3 4 5 6 7 |
| 22. I am able to utilize active interventions to help patients build up confidence to overcome disease and improve their quality of life. | 1 2 3 4 5 6 7 |
| 23. I have sufficient patience in responding to patients. | 1 2 3 4 5 6 7 |
| 1. I am able to mobilize patients' family members to provide encouragement and comfort to the patients. | 1 2 3 4 5 6 7 |
| 25. I am able to use treatment documents (such as letters, certificates, group photos, etc.) reasonably to give patients strength and courage. | 1 2 3 4 5 6 7 |
| 26. I am able to describe the patients story and their perceptions in plain language. | 1 2 3 4 5 6 7 |
| 27. I am able to enhance my identification and enthusiasm for the profession in the process of conducting narrative medicine. | 1 2 3 4 5 6 7 |
